# Potential survival benefit and early recovery from organ dysfunction with polymyxin B hemoperfusion: perspectives from a real-world big data analysis and the supporting mechanisms of action

**DOI:** 10.1186/s44158-022-00056-5

**Published:** 2022-06-20

**Authors:** Hisataka Shoji, Ricard Ferrer

**Affiliations:** 1 Division of Emergency and Critical Care Medicine, Toray Medical Co., Ltd, Tokyo, 103-0023 Japan; 2 Intensive Care Department, Vall d’Hebron University Hospital, SODIR Research Group, Vall d’Hebron Institut de Recerca, 08035 Barcelona, Spain

**Keywords:** Septic shock, Endotoxin adsorption, Polymyxin B, Hemoperfusion, Large-scale data set, Diagnosis procedure combination, Medical economy, Review

## Abstract

**Background:**

Endotoxin (ET) removal therapy with polymyxin B-immobilized fiber column hemoperfusion (PMX-HP) has been used for the treatment of septic shock. Some observational studies reported clinical benefits, particularly in specific subgroups of patients. However, larger randomized controlled trial results have been disappointing.

**Main body:**

The four studies that revealed the survival benefit of PMX-HP were based on the Japanese Diagnosis Procedure Combination (DPC) national inpatient database (J-DPC study). Nevertheless, one J-DPC study and a randomized controlled trial (RCT) conducted in France evaluated PMX-HP in patients with abdominal septic shock and did not report a significant survival benefit. In both studies, the severity of illness was too low to find substantial significant differences in mortality. The results of the J-DPC studies further suggest that some subpopulations of patients could benefit from PMX-HP. Based on these results, this review revisited prior RCTs and other large-scale studies on PMX-HP. In addition, four J-DPC studies and one large-scale study reported a survival benefit with PMX-HP. A secondary analysis of the EUPHRATES trial, the most recent double-blinded RCT of PMX-HP conducted in North America, suggested a survival benefit in patients with high levels of endotoxemia. In the J-DPC studies and the EUPHRATES trial, ventilator-free days, vasoactive drug-free days, and renal replacement-free days were significantly improved in the PMX-HP groups. These findings suggest that PMX-HP can contribute to early recovery from organ dysfunction. The reduction of supportive care likely provides important health and economic benefits for managing patients with septic shock. Finally, the blood levels of mediators or biomarkers related to respiratory, cardiovascular, and renal dysfunction have been reported to be normalized with PMX-HP.

**Conclusions:**

These results support the biological rationale for the improvement in organ dysfunction observed in the J-DPC studies and other large-scale studies, including the EUPHRATES trial. Real-world evidence from large data sets suggests an appropriate patient population that are likely to benefit from the utility of PMX-HP for septic shock.

## Background

Sepsis is a life-threatening complication caused by a dysregulated host response to infection. Sepsis and septic shock remain major healthcare problems. In 2017, there were 48.9 million incident cases of sepsis and 11.0 million sepsis-related deaths worldwide, representing 19.7% of all global deaths [1].

Over the years, endotoxin (ET) has been considered as an important therapeutic target for the treatment of sepsis and septic shock. Polymyxin B-immobilized fiber column hemoperfusion (PMX-HP) for ET removal is the most widely used blood purification therapy for sepsis. The PMX-HP procedure is practiced through whole blood circulation at a flow rate of 80–120 mL/min and lasts for 2 h with two scheduled sessions [2]. The effectiveness of this therapy has been documented in many published clinical articles [2, 3]. However, the associated survival benefit and improvement in organ dysfunction remain a subject of debate owing to the lack of definitive randomized controlled trials (RCTs).

Surviving Sepsis Campaign (SSC) guidelines 2016 made no recommendation regarding the use of blood purification techniques. In the recent SSC guidelines 2021, the panel issued a weak recommendation against the use of PMX-HP [4]. A meta-analysis of all available RCTs for PMX-HP reported a possible reduction in mortality (RR 0.87; 95% *CI* 0.77–0.98, low quality). However, this result was challenged by sensitivity analyses since after excluding trials with high risk of bias, the risk ratio was 1.14 (95% *CI* 0.96–1.36). Moreover, after excluding trials published before 2010, PMX-HP was found to be associated with a high mortality risk (*RR* 1.23; 95% *CI* 1.04–1.46). Overall, the quality of evidence is judged as low.

Recently, real-world evidence and large-scale data sets have become more widely accepted as complementary or alternative to RCTs. The Diagnosis Procedure Combination (DPC) database is an inpatient database that includes discharge data and administrative claim data. The hospitals are included in the DPC system in Japan based on a prospective payment system for inpatient medical treatment fee reimbursement scheme. As of April 2020, 1757 hospitals across Japan are part of the DPC system.

Studies investigating the effectiveness of PMX-HP treatment based on the Japanese DPC national inpatient database (J-DPC studies) have been recently conducted. The J-DPC database reflects real clinical experience in the country. The results of J-DPC studies suggest the patient population who are likely to benefit from PMX-HP treatment. Information on the effectiveness of PMX-HP extracted from the results of J-DPC studies helps us understand the reasons behind the challenges in obtaining statistically significant mortality differences in some of the previous PMX-HP studies, including the earlier RCTs.

The aim of this narrative review is to address the evidence on the effect of endotoxin removal with PMX-HP, especially its role on improving organ dysfunction and reducing mortality. We reviewed the results of a real-world big data analysis for PMX-HP and revisited the last large-scale clinical studies including RCTs. Moreover, we reviewed the studies assessing the immunomodulatory effects of PMX-HP to explain the mechanisms of action to support the observed improvements in respiratory, hemodynamic, and renal dysfunction in critically ill patients with septic shock.

### PMX-HP and mortality in patients with abdominal septic shock: evidence from RCTs and the J-DPC study

Abdominal septic shock is as an indication for PMX-HP since gram-negative bacterial infections are highly indicative of endotoxemia. The ABDOMIX group published the results of a prospective multicenter RCT performed in 18 French intensive care units (ICUs) between October 2010 and March 2013. This trial enrolled 243 patients with septic shock within 12 h after emergency surgery for peritonitis related to organ perforation [5]. However, only 81 patients in this trial completed the two sessions of PMX-HP. The results showed that the 28-day mortality in the PMX-HP group (*n* = 119) was 27.7%, whereas that in the conventional group was 19.5% (*n* = 113) (*p* = 0.14, *OR*: 1.5872, 95% *CI*: 0.8583–2.935). Hence, based on this multicenter RCT, PMX-HP resulted in a nonsignificant increase in mortality and non-improvement in organ dysfunction compared with conventional treatment of peritonitis-induced septic shock.

Nevertheless, real-world evidence and studies with large sample sizes have shown different results. For example, Iwagami et al. examined the effect of postoperative PMX-HP on mortality in patients with abdominal septic shock triggered by lower gastrointestinal tract perforation using the J-DPC database [6]. Patients aged ≥ 18 years old who were hospitalized between July 2007 and October 2011 were included. Of the 2925 eligible patients, 642 received one or two PMX-HP sessions, where the first session was started on day 0 (day of admission) or day 1. Propensity score matching created a matched cohort of 1180 patients (590 pairs with and without PMX-HP). The 28-day mortality was 17.1% (101/ 590) in the PMX-HP group and 16.3% (96/ 590) in the control group *(p*= 0.696). Thus, no significant improvement in 28-day mortality was reported. However, the overall mortality rate of the control group in both the ABDOMIX (19.5%) and J-DPC studies (16.3%) was low compared with that of similar patient cohorts [7].

Iwagami et al. further studied the survival benefit of PMX-HP in patients with septic shock requiring continuous renal replacement therapy (CRRT) for acute kidney injury (AKI), who are known to have an increased risk of mortality [8]. Adult patients in the J-DPC database satisfying the following criteria were enrolled: hospitalization between 2007 and 2012, diagnosis of sepsis, noradrenaline or dopamine infusion requirements, and initiation of CRRT in the ICU. Of the 3759 eligible patients, 1068 received PMX-HP, and 2691 did not. Propensity-score matching produced a matched cohort of 978 pairs. In the subgroup analyses of this cohort, PMX-HP reduced the 28-day mortality (PMX-HP group: 35.8% (182/508) vs. control group: 44.0% (229/521), *OR*: 0.71, 95% *CI*: 0.55–0.92) in patients with abdominal infection most likely caused by gram-negative bacteria. Although the survival benefit with PMX-HP was not observed in their earlier study [6], it became evident once a sicker patient population on CRRT with abdominal septic shock was analyzed.

Consequently, it is hypothesized that severely affected patients, who have the highest likelihood to benefit from PMX-HP, may not have been included in Iwagami et al’s [6]. and ABDOMIX studies [5].

### Effects of PMX-HP: evidence from J-DPC studies

#### Improvement in survival rate and organ dysfunction

The clinical benefits of PMX-HP in patients with sepsis requiring CRRT have been a topic of interest [9]. Based on the 2007–2012 Japanese databases, Iwagami et al. demonstrated the survival benefit of PMX-HP in patients with septic shock requiring CRRT owing to AKI [8]. The 28-day mortality for the propensity-matched groups was significantly lower in the PMX-HP group than the control group (PMX-HP group: 40.2% (393/978) vs. control group: 46.8% (458/978), *p* =0.003) [8]. Fujimori et al. identified 17,367 adult patients with sepsis who received continuous hemodiafiltration (CHDF) with or without PMX-HP from the J-DPC database between April 2016 and March 2019. The number of patients who received CHDF was 12,748 [10]. Among these patients who received CHDF, 8222 (53.5% of the total population) were also treated with PMX-HP, and 4526 (29.5% of the total) were not. After propensity score matching, 3751 patient pairs were generated. Mortality at 28 days was significantly lower in the CHDF + PMX-HP group than in the CHDF-only group (30.5% vs. 34.6%, *p* < 0.0001), with a hazard ratio of 0.905 (95% *CI*: 0.875–0.936). This result was similar to that of Iwagami et al.; however, the mortality rate was lower compared with the findings in Iwagami et al. [8].

Furthermore, Fujimori et al. reported a significantly shorter length of hospital stay in the CHDF + PMX-HP group than in the CHDF-only group (*p* < 0.0001, log-rank test). Regarding the ICU and emergency room (ER) length of stay, the median stay was 9 (interquartile range [IQR]: 5–13) days in the CHDF + PMX-HP group and 11 (IQR: 6–13) days in the CHDF-only group (*p* = 0.0016). The reduction in the duration of ICU and emergency room stay is beneficial to patients and likely reduces the cost of medical care.

The effects of PMX-HP on patients with septic shock requiring noradrenaline infusion were also analyzed using the J-DPC database between April 2016 and March 2019 [11]. A total of 30,731 adult patients with septic shock treated with noradrenaline met eligibility criteria. Propensity score matching produced a matched cohort of 4141 pairs in the PMX-HP group and control group. The 28-day mortality rate was 22.1% in the PMX-HP group and 28.9% in the control group (*p* < 0.0001, *OR*: 1.433, 95% *CI*: 1.298–1.584). In the matched groups, CHDF was used in 2460 (59.4 %) patients in the PMX-HP group and 2549 (61.6%) in the control group. Mechanical ventilation was used in 2961 (71.5%) patients in the PMX-HP group and 3073 (74.2%) patients in the control group. The number of patients who used CHDF and mechanical ventilation did not significantly differ between the PMX-HP and the control groups. The number of noradrenaline-free days, CHDF-free days, and ventilation-free days was significantly higher in the PMX-HP group than in the control group, with a median difference of 2 days (*p* < 0.0001), 2 days (*p* < 0.0001), and 6 days (*p* < 0.0001), respectively.

Since 2018, in the J-DPC data, patients diagnosed with sepsis have their Sequential Organ Failure Assessment (SOFA) score assessed during the first 48 h after diagnosis. Fujimori et al. examined the association between SOFA score at the onset of sepsis and the efficacy of PMX-HP treatment using the J-DPC database between April 2018 and March 2020 [12]. During the study period, 74,879 patients met the inclusion criteria. Among these, 30,702 patients were excluded because of missing data; thus, 44,177 patients were finally included in the study. There were 2191 patients who received PMX-HP and 41,986 patients who did not. After propensity score matching, 2033 patient pairs were created. In both patient groups with SOFA scores ranging from 7–9 to 10–12, the 28-day mortality was significantly lower in the PMX-HP group than in the control group (Table 1). There were no significant differences in the mortality rate between the groups with SOFA scores ranging from 0–6, 13–15, or 16–24.

**Table 1:** Differences in the mortality rate and noradrenaline-free days between the patients in the PMX-HP-treated group and the control group stratified by SOFA score

	28-day mortality range (%) Fatality/the number of patients (***n***)			Noradrenaline-free days Median (IQR)		
SOFA score range	PMX-HP group	Control group	***p***-value	PMX-HP group	Control group	***p***-value
**0–6**	15.0–15.2 (69/456)	9.1–16.7 (47/407)	NS	25 (21-–6)	25 (20–26)	0.9287
**7–9**	14.2–16.1 (83/553)	16.2–22.9 (92/463)	0.0410	25 (20–26)	24 (11–26)	0.0003
**10–12**	14.8–25.3 (95/510)	25.3–30.6 (145/529)	0.0008	24 (15–26)	22(0–6)	0.0005
**13–15**	28.5–39.3 (116/356)	24.8–35.5 (118/404)	NS	22 (0–25)	21.5 (0–25)	0.9161
**16–24**	30.6–61.5 (62/158)	34.6–57.1 (96/230)	NS	14 (0–24)	15 (0–4)	0.3491

Twenty-eight-day mortality range represents the minimum and the maximum number. IQR denotes interquartile range and NS not significant

Regarding organ-support-free days, ventilator-free days were considerably higher in the PMX-HP group with SOFA scores ranging from 7–9 to 10–12. Noradrenaline-free days were significantly higher in the PMX-HP group with SOFA scores ranging from 7–9 to 10–12. CHDF-free days were higher in the PMX-HP group with SOFA scores ranging from 0–6, 7–9, and 10–12. These results indicate that reduced mortality and morbidity from PMX-HP treatment could not be expected in the most severely ill patients with a SOFA score > 12. Fujimori et al.’s findings suggest that the efficacy of PMX-HP is more pronounced in patients with moderately elevated SOFA scores ranging from 7 to 12. The averaged mortality rate of the control group with SOFA scores between 7 and 9 was 19.9%. In Iwagami et al.’s J-DPC study [6], the mortality rate of the control group was low (16.3%). Thus, the lower mortality in the control group is attributed to the lack of benefit from PMX-HP treatment observed in this study. In contrast, the mortality rate of the control group in the ABDOMIX study was 19.5%, which is comparable to the mortality rate of 19.9% in patients with SOFA scores ranging from 7 to 9 as reported by Fujimori et al. [12]. However, the ABDOMIX study did not demonstrate a survival benefit. Thus, PMX-HP treatment may be the most effective in reducing sepsis-related mortality and morbidity in severely ill patients with a non-imminent death state.

### Effects of PMX-HP: evidence from RCTs

#### Improvement in organ dysfunction and survival rate

The multicenter, randomized, double-blinded, sham-controlled EUPHRATES trial aimed to determine whether adding PMX-HP to conventional medical therapy improves survival in comparison with conventional therapy among patients with septic shock and high ET activity, defined as an ET activity (EA) value ≥ 0.60 as measured by ET activity assay [13]. No difference in 28-day all-cause mortality (PMX-HP group, 84/223 (37.7%), and control group 78/226 (34.5%), *p* = 0.49) was observed in the patients with a multiple organ dysfunction score (MODS) of > 9 (PMX-HP group, 65/146 (44.5%), control group 65 /148 (43.9%), *p* = 0.92). Regarding the secondary and exploratory endpoints, the increase in mean arterial pressure (MAP) at day 3 was significantly higher in the PMX-HP group than in the control group (difference: 5.5 mmHg, 95% *CI*: 2.5–8.6, *p* < 0.005) in patients with a MODS of > 9.0 (difference: 4.5 mmHg, 95% *CI*: 0–8.3, *p* = 0.02). Ventilation-free days at day 28 were also significantly higher in the PMX-HP group than in the control group in patients with a MODS score of > 9.0 (difference: 2.9, 95% *CI*: 0.5–5.3, *p* = 0.02).

In a post hoc analysis of the EUPHRATES trial, Klein et al. evaluated the effect of PMX-HP use in patients with septic shock with moderately severe disease and non-moribund condition, as defined as a high severity of illness (MOD score > 9) and an EA value ranging from 0.6 to 0.89 [14]. At 28 days, 23 of 88 (26.1%) patients in the PMX-HP group died, whereas 39 of 106 (36.8%) patients in the control group died (risk difference: 10.7%, *OR*: 0.52, 95% *CI*: 0.27–0.99, *p* = 0.047). Kaplan–Meier analysis revealed that the 28-day survival is higher in the PMX-HP group than the control (*HR* 0.56, 95% *CI*: 0.33–0.95, *p* = 0.03). PMX-HP patients had a greater increase in MAP (median [IQR] 8 mmHg [−0.5, 19.5] vs. 4 mmHg [−4.0, 11], *p* = 0.04) and a higher number of ventilation-free days (median [IQR] 20 days [0.5, 23.5] vs. 6 days [0, 20], *p* = 0.004) than those in the control group. However, a nonsignificant trend in the median number of days alive and free of dialysis (20 days vs. 11 days; *p* = 0.59) and in the length of hospital stay (PMX-HP, 22.0 days vs. control 28 days; *p* = 0.15) was observed between both groups. As a follow-up of the EUPHRATES trial, the TIGRIS (Toraymyxin use to Information Gather Regarding Its efficacy and Safety for patients with endotoxemic septic shock) trial is currently ongoing in the USA and aims to validate their findings [15].

Nakamura et al. performed a retrospective analysis of the Japan Septic Disseminated Intravascular Coagulation (JSEPTIC DIC) study database [16] and included 1723 patients with septic shock aged ≥ 16 years. Patients were stratified into the PMX-HP and non-PMX-HP groups by propensity score matching, and 262 matched pairs were generated. A nonsignificant difference in the use of CRRT in 121 of 262 (46.2%) patients in the non-PMX-HP group and 124 of 262 (47.3%) patients in the PMX-HP group was observed. The mean SOFA score was 11.7 (standard deviation: 3.4) in the non-PMX-HP group and 11.5 (3.4) in the PMX-HP group. After propensity score matching, the baseline patient characteristics were well-balanced between the two groups. The all-cause hospital mortality rate was significantly lower in the PMX-HP group than in the non-PMX-HP group (32.8% vs. 41.2%; *OR*: 0.681; 95% *CI*: 0.470–0.987; *p* = 0.042). The number of ICU-free days in the first 28 days was significantly longer in the PMX-HP group than in the non-PMX-HP group (18 days, 95% *CI*: 0–22 vs. 14 days, 95% *CI*: 0–22, respectively; *p* = 0.045). The SOFA score of the matched pairs from the JSEPTIC DIC study was approximately 11 in both groups. Considering the results of the J-DPC study by Fujimori et al. [12], the severity of the patients enrolled in was hypothesized to be adequately sufficient to obtain a significantly improved clinical outcome in the PMX-HP-treated group. The outcomes of the J-DPC studies and other large-scale studies are summarized in Table 2.

**Table 2:** Comparisons of the outcomes between PMX-HP and control group in the J-DPC studies and some large-scale studies including RCTs

	Length of ICU/R stay (days)/HP stay rate at day 28(%)	MV-free days	NAD-free days/increasing of BP	CHDF-free days	28-day outcome (%)
**Iwagami et al.** **[**8**]**	NA	NA	NA	NA	(Mortality) 40.2 vs 46 *OR* 0.76 *p* = 0.003
**Fujimori et al.** **[**11**]**	NA	20 (1–28) vs 14 (0–28) *p* < 0.0001	24 (11–26) vs 22 (0–25) *p* < 0.0001	24 (9–28) vs 22 (0–28) *p* < 0.0001	(Survival rate) 77.9 vs 71.1 *OR* 1.433 *p* < 0.0001
**Fujimori et al.** **[**10**]**	9 (5–13) vs 11 (6–13), *p* = 0.016**/**81.6 vs 3.4% *HR* 1.083	NA	NA	NA	(Survival rate) 69.5 vs 65.4 *HR* 0.905 *p* < 0.0001
**Fujimori et al.** **[**12**]**	NA	16 (0–23) vs 12 (0–22) *p* = 0.0061	24 (15-26) vs 22(0-26) *p* = 0.0005	23 (8–25) vs 21 (0–24) *p* = 0.0034	(Mortality) 18.6 vs 27.4 *p* = 0.0008
**Dellinger, R. P. et al.** **[**13**]**	**/**NS	mean (SD) 12.7 (10.9) vs 9.8 (10.0) *p* = 0.02	mean (SD) 8.1 (16.0) vs 3.9 (14.1), *p* = 0.02**/**	NS	NS
**Klein, D. J. et al.** **[**14**]**	**/**NS	20 (0.5–23.5) vs 6 (0–20) *p* = 0.004	**/**8 (−0.5–19.5) vs 4 (−4.0–11) *p* < 0.05	NS	(Mortality) 26.1 vs 36.8, *OR* 0.52, *p* = 0.047
**Nakamura, Y. et al.** **[**16**]**	14 (0–22) vs 18 (0–22) *p* < 0.045**/**	NA	NA	NA	(HP mortality) 32.8 vs 41.2 *OR* 0.681 *p* = 0.042

Data is represented as the comparison between PMX-HP-treated group and control group (PMX-HP versus control). Data from the reference [12] study refers to SOFA score range 10–12.

- Length of ICU/ER stay and/or hospital stay rate: Data is represented as median (IQR).
- MV-free days: Data is represented as median (IQR) except for the reference [13] study in which the data is represented as median (SD).
- NAD-free days/increasing of BP: Data is represented as median (IQR) except for the reference [13] study in which the data is represented as mean (SD).
- CHDF-free days: Data is represented as median (IQR).
- Twenty-eight-day or hospital outcome: Either survival rate or mortality is shown.

The difference is described in each parenthesis. NS denotes not significant, NA not applicable, IQR interquartile range, SD standard deviation, OR odds ratio, MV mechanical ventilation, NAD noradrenaline, CHDF continuous hemodiafiltration, and HP hospital.

### Differences between RCTs, large observational studies, and meta-analyses

Clinical studies evaluating the efficacy of PMX-HP include RCTs, observational studies using large registry data, and meta-analyses. As a study to evaluate the efficacy of a treatment, RCTs have the great advantage of being free from concerns of bias in the grouping of patients, since they randomly assign the patients with or without treatment. On the other hand, it is widely recognized that it is difficult to enroll a large number of patients in a short period of time, especially in critical care field such as sepsis, and it is difficult to show a significant difference in mortality [17, 18]. For example, assuming survival rates of 50% and 45% for the two groups, the number of samples needed to obtain a significant difference in the mortality is estimated to be about 3000, which is unrealistic for a study on sepsis, especially a study on medical devices. The largest RCT of PMX-HP, the EUPHRATES trial, included 450 patients [13].

Observational studies using registry data have the challenge that even when patient background is adjusted by using methods such as propensity score matching, the possibility that unadjusted confounding factors may exist and cause bias in the results cannot be eliminated. On the other hand, the number of data that can be analyzed is much larger in observational studies compared to RCTs. The J-DPC study described above, which examined the efficacy of PMX-HP in patients with septic shock treated with noradrenaline, included 8282 patients in the analysis and was able to detect a 6.8% mortality difference with a significant difference [11]. The analysis of registry data also has the advantage of providing results that reflect real-world clinical practice, unlike RCTs that only enroll patients who meet specific criteria. The results of RCTs and observational studies should be used in a complementary manner to evaluate the effectiveness of treatments [17].

A meta-analysis that integrates and evaluates multiple RCTs is considered a study that provides the highest level of evidence and is employed as an evaluation to determine recommendations in many guidelines. However, the results of meta-analyses can vary widely depending on how the articles to be evaluated are selected, and the results of a single meta-analysis do not always provide reliable evidence. In fact, nine meta-analyses evaluating the efficacy of PMX-HP have been reported so far, but the papers that included in each meta-analysis are different. As a result, six studies among nine [19–24] found a significant survival benefit of PMX-HP, while other three studies [25–27] found no such benefit with statistically significant levels.

### Organ dysfunction improvement in patients treated with PMX-HP and the supporting mechanisms of action

Mechanistically, ET adsorption is the fundamental mechanism of action of PMX-HP. A possible secondary mechanism is the adsorption of immune cells, such as activated neutrophils and monocytes [3, 28]. The polymyxin B molecule has a strong affinity to ET through ionic and hydrophobic interactions. The negatively charged phosphate groups in the lipid A portion of ET interact with the positively charged primary amino groups of α, γ-diaminobutyric acid residues of polymyxin B. Hydrophobic interactions occur between the fatty acid chains of the lipid A portion and the hydrophobic amino acids and methyloctanoic acid in the polymyxin B molecule.

Polymyxin B-immobilized fiber (PMX-F) is a selective adsorbent of ET [29]. The adsorbents can bind substances in the blood via ionic and hydrophobic bindings, such as cytokines and humoral mediators of systemic inflammation. Utsunomiya et al. studied cytokine adsorption by PMX-F in vitro [30] and reported that PMX-F could adsorb various cytokines associated with inflammation, fibrosis, and vascular permeability, including interleukin (IL)-1β, IL-6, IL-8, RANTES (regulated on activation, normal T cell expressed and secreted), MCP-1 (monocyte chemoattractant protein-1), FGF2 (fibroblast growth factor-2), PDGF-bb (platelet-derived growth factor-BB), TGF-β (transforming growth factor-β), and VEGF (vascular endothelial growth factor). The authors attributed the beneficial effects of PMX-HP on pulmonary oxygenation and prognosis in patients with acute exacerbation of interstitial pulmonary fibrosis (AE-IPF) to removal of multiple cytokines. Thus, cytokine hemadsorption with PMX-HP may significantly reduce inflammation and improve organ dysfunction.

### Effect of PMX-HP on pulmonary oxygenation

Many studies reported an increase in the PaO_2_/FiO_2_ ratio after PMX-HP treatment. Activated neutrophils migrate into the alveoli and damage lung epithelial cells. Kushi et al. studied the relationship between the changes in inflammatory mediators and the PaO_2_/FiO_2_ ratio in 36 patients with septic shock (21 men, mean age: 62 ± 18.5 years) complicated with acute lung injury or acute respiratory distress syndrome (ARDS) treated with PMX-HP [31]. The authors evaluated the changes in IL-8 levels in the blood as an index of neutrophil activation, plasminogen activator inhibitor-1 (PAI-1) as a marker of the blood endothelial cell injury, and peripheral blood neutrophil elastase (NE) as a mediator of neutrophil-associated injury. Following the initiation of PMX-HP, the mean blood levels of IL-8, PAI-1, and NE became significantly lower after 48 h, and the PaO_2_/FiO_2_ ratio significantly increased (from 244 ± 26.3 mmHg to 322 ± 22.1 mmHg, *p* < 0.05) after 96 h of treatment in PMX-HP patients. The PaO_2_/FiO_2_ ratio was inversely correlated with blood NE (correlation coefficient between the log-transformed PaO_2_/FiO_2_ ratio and log-transformed NE value: −0.337*, p* = 0.0198) and IL-8 (correlation coefficient between the log-transformed PaO_2_/FiO_2_ ratio and log-transformed IL-8 value: −0.417, *p* = 0.0032) levels. They concluded that the improvement in PaO_2_/FiO_2_ ratio is related to the reduction in the levels of humoral mediators due to ET removal.

Ishibe et al. found that the plasma levels of type II secretory phospholipase A2 (sPLA2-II) and surfactant protein-D (SP-D) in patients with septic ARDS are simultaneously elevated with inflammatory cytokine levels (e.g., TNF-α and IL-8) [32]. sPLA2-II, excreted into the alveoli by macrophages and mast cells, plays an important role in the development of respiratory dysfunction. Surfactant disruptions can also cause lung disorders. The authors studied the association of sPLA2-II and SP-D with decreases in pulmonary oxygenation indices in patients with septic shock treated with PMX-HP. A total of 25 patients with septic shock with ARDS (16 men, mean age: 71.9 ± 7.8 years, SOFA score 12.1 ± 4.1) were enrolled. Blood ET levels were significantly reduced from 12.4 ± 23.5 pg/mL (before the first treatment session) to 0.9 ± 1.0 pg/mL a day following treatment. The plasma TNF-α levels on day 0 were 183.6 ± 120.6 pg/mL and decreased to 69.7 ± 44.0 pg/mL on day 2. From day 0 (immediately before the 1st PMX-HP session) to day 2, the plasma sPLA2-II levels significantly decreased from 340.0 ± 150.7 ng/mL to 189.0 ± 73.4 ng/mL (*p* < 0.05), and the plasma SP-D levels decreased from 483.3 ± 290.0 ng/mL to 251.6 ± 117.0 ng/mL (*p* < 0.05). The PaO_2_/FiO_2_ ratio significantly increased from 210.0 ± 51.8 mmHg on day 0 to 262.2 ± 52.1 mmHg on day 2 (*p* < 0.05). There was a positive correlation between the plasma sPLA2-II levels and the plasma SP-D levels on day 0 (before PMX-HP initiation) (*r* = 0.89, *p* < 0.05). However, there was an inverse correlation between the plasma sPLA2-II levels and the PaO_2_/FiO_2_ ratio on day 0 (before PMX-HP initiation) (*r* = 0.72, *p* < 0.05) and between the plasma SP-D levels and the PaO_2_/FiO_2_ ratio on day 0 (*r* = 0.87, *p* < 0.05). They speculated that inflammatory reactions were suppressed following ET removal by PMX-HP, thereby preventing the formation of TNF-α, PLA2-II, and SP-D and improving pulmonary oxygenation.

### Effect of PMX-HP on hemodynamics

Improving hemodynamics (increased blood pressure and reduced vasopressor requirements) in septic shock patients is the most clinically evident effect associated with PMX-HP treatment [33–35]. The EUPHAS (early use of polymyxin B hemoperfusion in abdominal septic shock) randomized controlled trial [7] observed an increase in MAP (from 76 to 84 mm Hg; *p* = 0.001) and a decrease in vasopressor requirements (inotropic score, 29.9 to 6.8; *p* < 0.001) at 72 h in the PMX-HP group but not in the control group (MAP, from 74 to 77 mm Hg; *p* = 0.37; inotropic score, 28.6 to 22.4; *p* = 0.14). Both the EUPHRATES trial [13] and a post hoc analysis [14] reported a significant improvement in MAP in the PMX-HP groups compared with control groups.

Sugiura et al. retrospectively investigated 78 consecutive patients with severe sepsis or septic shock who received PMX-HP [36]. They classified the patients into two groups based on inotropic score improvement after PMX-HP as follows: improvement group and non-improvement group. Patient characteristics, such as SOFA score, blood ET levels, causative organisms, status of immunosuppressive conditions, and other variables, did not significantly differ between the two groups. However, the inotropic score prior to PMX-HP treatment was significantly higher in the improvement group than in the non-improvement group (*p* < 0.01). The positive change in the PaO_2_/FiO_2_ ratio following PMX-HP was also significant in the improvement group (*p* < 0.05). Hence, PMX-HP was suggested to be particularly useful for improving hemodynamics in patients with septic shock.

In a single-arm clinical trial of 37 patients with sepsis having endotoxemia and treated with PMX-HP, Kodama et al. reported improved cardiovascular parameters [37]. These patients received 51 PMX-HP sessions in total. Hemodynamic parameters were monitored with a Swan-Ganz catheter. In the evaluated 17 sessions of PMX-HP, the patients had initial systemic vascular resistance index (SVRI) of ≥ 900 (dyne·s·cm^−5^; mean value 638 ± 37 dyne·s·cm^−5^) before treatment. After PMX-HP sessions, the SVRI significantly increased to 717 ± 54 immediately after PMX-HP (*p* < 0.05) and further increased to 773 ± 49 on the following day (*p* < 0.01). In a pilot study, Vincent et al. reported that the patients treated with PMX-HP had significant increases in cardiac index (CI; *p* = 0.012 and 0.032 at days 1 and 2, respectively), left ventricular stroke work index (*p* = 0.015 at day 2), and oxygen delivery index (*p* = 0.007 at day 2) compared with controls [38].

Nakamura et al. studied the changes in the plasma levels of atrial natriuretic peptide (ANP) and brain natriuretic peptide (BNP) in septic shock patients treated with PMX-HP [39]. These levels are markedly elevated in patients with septic shock. Increased plasma ANP and BNP levels as predictors of cardiac dysfunction were observed. The plasma levels of ET, IL-6, ANP, and BNP were significantly increased in patients with septic shock (*n* = 50) compared with healthy control subjects (*n* = 30), significantly decreased after two sessions of PMX-HP, and further reduced the following day. The cardiac ejection fraction increased significantly after PMX-HP. These results suggest that ET removal may be a good therapeutic target to prevent myocardial injury in patients with septic shock.

The direct removal of vasodilatory mediators using PMX-HP may be another mechanism underlying the rapid improvements in blood pressure in patients with septic shock. Anandamide (ANA), an endogenous cannabinoid, can be produced by activated macrophages during shock with endotoxemia. This molecule is thought to be a paracrine contributor to hypotension. Wang et al. revealed that ANA is efficiently adsorbed in polymyxin B-immobilized beads [40], suggesting that PMX-HP could directly remove ANA molecules in patients with septic shock and subsequently increase blood pressure.

### Effect of PMX-HP on acute kidney injury

Fujimori et al. analyzed 17,367 patients diagnosed with sepsis in the J-DPC database between April 2016 and March 2019 [10]. After excluding those aged < 20 years and those who died within 24 h after admission or within 2 days from the trigger date (defined as the day when either CHDF or PMX-HP was started), there were 15,364 eligible patients, 83% (12,748) of which received CHDF treatment. CHDF is frequently used for fluid management in patients with septic shock having AKI for the treatment of severe shock, renal support, and removal of inflammatory mediators.

Podocytes are located on the outer surface of the glomerular basement membrane and play an important role in glomerular filtration. Detection of podocytes in the urine sediment indicates severe injury in pediatric renal disease. Some studies have suggested that ET causes direct injury of renal cells. Shimada et al. studied the detection of podocytes in the urine of severe sepsis patients with AKI and assessed how ET removal with PMX-HP affects the number of urinary podocytes [41]. They studied 20 patients with sepsis (mean age: 44.6 years old, range: 26–58) and 20 healthy controls (mean age: 41.6 years old, range: 28–54). Urinary podocytes were detected in 12 of 20 patients with sepsis. The number of podocytes decreased from 2.8 ± 0.8 cells/mL to 0.6 ± 0.4 cells/mL following the reduction in plasma ET levels (from 38.8 ± 9.8 to 3.6 ± 0.6 pg/mL) after PMX-HP treatment. Thus, the number of urinary podocytes might be a good marker of sepsis-induced renal injury. This study showed that PMX-HP effectively reduces the number of urinary podocytes by removing ET in blood.

Netti et al. studied the therapeutic efficacy of ET removal in decreasing albuminuria by reducing podocyte CD80 expression [42]. An increase in CD80 expression in podocytes was reported in several proteinuric glomerulopathies and associated with worse renal outcomes. Selective ET removal (with coupled plasma filtration and adsorption (CPFA) system or PMX-HP) was demonstrated to reduce blood ET level (EA values) and decrease proteinuria, CD80 expression, and urinary excretion in both animal and clinical models.

Cantaluppi et al. reported that PMX-HP decreased the proapoptotic activity of the plasma of patients with sepsis on cultured renal cells and found a strong correlation between the reduced levels of blood ET and plasma-induced tubular apoptosis [43]. By removing ET from the blood, the proapoptotic activity of the plasma of patients with sepsis was reduced. Mitaka et al. also reported that PMX-HP therapy might protect against AKI by inhibiting the NF-kβ signaling pathway and preventing renal tubular cell apoptosis in a rat model [44]. Reducing the levels of proapoptotic factors using PMX-HP by removing ET or direct adsorption of proapoptotic factors could be useful for the early prevention of AKI.

**Fig. 1:**
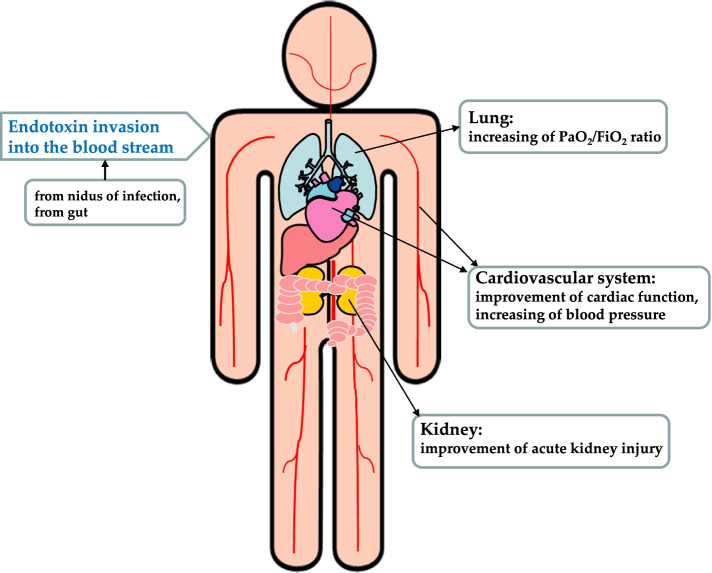
Improvement of organ dysfunction for septic shock patients with endotoxemia accompanied with PMX-HP

Improvement of organ dysfunction accompanied with PMX-HP is illustrated to summarize the abovementioned data (Fig. 1).

Effectiveness of endotoxin removal with PMX-HP has been indicated through clinical application for more than a quarter of a century. The appropriate use of PMX-HP is still required to exert an anticipated clinical effectiveness. PMX-HP should be conducted adequately for the right patients by the right timing. The selected subgroups of septic shock patients with endotoxemia and the proper level of severity of illness could benefit from this treatment. PMX-HP should be started as soon as possible if the patients fulfil the abovementioned condition. The number of sessions required for PMX-HP is one of the future directions to be tailored depending on each patient’s response.

Recently, the use of PMX-HP for severe COVID-19 patients has been reported [45, 46]. It is well recognized that overwhelming cytokine production is a typical pathophysiology of severe COVID-19. Cytokine storm induces vascular endothelial cell injury and blood coagulation abnormalities, which progress to organ dysfunction such as ARDS, cardiovascular dysfunction, and AKI. Endotoxemia is suspected to be highly involved by the secondary gram-negative bacterial infection and/or endotoxin translocation from the gut due to the disturbance of gut barrier function. So, the potential role of endotoxin removal with PMX-HP for severe COVID-19 needs to be considered.

## Conclusions

The findings of recent studies based on real-world evidence and large-scale data sets, such as the J-DPC database, demonstrated the survival benefit of PMX-HP treatment. An increased in the number of ventilation-free days, CHDF-free days, and NAD-free days was observed in patients treated with PMX-HP, which supports previous reports that reported improvements in organ dysfunction in patients with septic shock treated with PMX-HP. These studies also provide important insights into the patient population who are likely to benefit from PMX-HP. Early improvements in organ dysfunction (e.g., respiratory, cardiovascular, and renal dysfunction) support the health and economic benefits of PMX-HP treatment for sepsis. Mechanistically, the changes in the blood levels of biomarkers related to organ dysfunction and improvement in cardiac function parameters associated with PMX-HP therapy provide evidence of the biological plausibility of the clinical observations reported in J-DPC and other studies. Continued analysis of accumulated large-scale data sets is required to obtain real-world evidence on the usefulness of PMX-HP for treatment of septic shock. The results of the TIGRIS trial and future studies will contribute to our understanding of this meaningful intervention.

## Data Availability

Not applicable
